# *Gnathostoma spinigerum* in Live Asian Swamp Eels (*Monopterus* spp.) from Food Markets and Wild Populations, United States

**DOI:** 10.3201/eid2004.131566

**Published:** 2014-04

**Authors:** Rebecca A. Cole, Anindo Choudhury, Leo G. Nico, Kathryn M. Griffin

**Affiliations:** US Geological Survey–National Wildlife Health Center, Madison, Wisconsin, USA (R.A. Cole, K.M. Griffin);; St. Norbert College, DePere, Wisconsin, USA (A. Choudhury);; US Geological Survey–Southeast Ecological Science Center, Gainesville, Florida, USA (L.G. Nico)

**Keywords:** Gnathostoma spinigerum, G. turgidum, G. lamothei, Monopterus albus, M. (Amphipnous) cuchia, Asian swamp eels, Synbranchidae, human gnathostomiasis, introduced species, Florida, Georgia, New Jersey, New York, parasites, foodborne diseases, Suggested citation for this article: Cole RA, Choudhury A, Nico LG, Griffin KM. *Gnathostoma spinigerum* in live Asian swamp eels (*Monopterus* spp.) from food markets and wild populations, United States. Emerg Infect Dis [Internet]. 2014 Apr [date cited]. http://dx.doi.org/10.3201/eid2004.131566

## Abstract

In Southeast Asia, swamp eels (Synbranchidae: *Monopterus* spp.) are a common source of human gnathostomiasis, a foodborne zoonosis caused by advanced third-stage larvae (AL3) of *Gnathostoma* spp. nematodes. Live Asian swamp eels are imported to US ethnic food markets, and wild populations exist in several states. To determine whether these eels are infected, we examined 47 eels from markets and 67 wild-caught specimens. Nematodes were identified by morphologic features and ribosomal intergenic transcribed spacer–2 gene sequencing. Thirteen (27.7%) *M. cuchia* eels from markets were infected with 36 live *G. spinigerum* AL3: 21 (58.3%) in liver; 7 (19.4%) in muscle; 5 (13.8%) in gastrointestinal tract, and 3 (8.3%) in kidneys. Three (4.5%) wild-caught *M. albus* eels were infected with 5 *G. turgidum* AL3 in muscle, and 1 *G. lamothei* AL3 was found in a kidney (both North American spp.). Imported live eels are a potential source of human gnathostomiasis in the United States.

In parts of Asia, wild-caught and aquaculture-reared swamp eels (Synbranchidae: *Monopterus* spp.) are widely consumed as food by humans (*1*–*3*) and are a common source of human gnathostomiasis, a foodborne zoonosis caused by advanced third-stage larvae (AL3) of *Gnathostoma* spp. nematodes. (*4*–*8*). Over the past 2 decades, many thousands of swamp eels (Synbranchidae: *Monopterus* spp.) have been legally shipped alive from Asia to North America, where they were distributed to numerous ethnic food markets in major cities in the United States and Canada (*9*; L.G. Nico, unpub. data). An earlier survey of live Asian swamp eels from ethnic markets in the United States and introduced wild populations in Florida found substantial parasite burden in both market and wild swamp eels sampled; however, the researchers did not examine eels for *Gnathostoma* spp. (*9*).

In US ethnic food markets, imported swamp eels from Asia, together with a variety of other native and nonnative fishes, are commonly displayed alive. Consumers are able to purchase the animals and have them processed on site (gutted/filleted) or they can butcher their live purchase at home (*9*). Most of these market fish are purchased for food, but some are introduced into the wild. For instance, in Asia and certain western countries, several live fish and other animals sold in food markets and other venues are subsequently released into open waters by groups conducting ceremonial religious practices (*10*–*12*) with some releases that apparently involved swamp eels (*9**,**13*; L.G. Nico, unpub. data). Because a large number of fishborne parasitic zoonoses are found throughout the world (*14*,*15*), the importation of live fish infected with parasites from their native waters poses a threat to humans (*14*,*16*). Moreover, releasing imported foreign fish infected with parasites into open waters may introduce and spread nonnative parasites harmful to native faunas (*17*,*18*).

Swamp eels are a group of eel-like percomorph fishes naturally distributed in tropical and temperate regions of the New and Old Worlds (*19*). They are not native to the United States or Canada, but at least 5 separate introduced populations of Asian swamp eels (*Monopterus* spp.) have been established in open waters in the continental United States. These consist of 3 populations in peninsular Florida, 1 in northern Georgia, and 1 most recently established population in southern New Jersey (*9*,*20*). The live food trade is the suspected source of all or many of these introductions (*9*). Genetic analysis revealed that the introduced wild populations are composed of 3 genetically distinct clades within the *M. albus* (Zuiew, 1793) complex, a widely-distributed group native to eastern and southeastern Asia (*20*,*21*). A separate Asian swamp eel species, *M. cuchia* (Hamilton-Buchanan, 1822), also referred to as *Amphipnous cuchia*, is native to northern and northeastern India, Bangladesh, Nepal, Myanmar, and Pakistan (*22*). *M. cuchia* and members of the *M. albus* complex have been documented in animals in the live food trade and in ethnic food markets in the USA, but *M. cuchia* has not yet been documented in the United States in wild populations (L.G. Nico, unpub. data). All swamp eel species sold in the live food trade have behavioral and physiologic adaptations that make them attractive for live import and increase the risk for their invasion success in the wild. For example, both *M. albus* and *M. cuchia* eels are air breathers and, if kept moist, they can survive for months out of water and without food (*23*; L.G. Nico, unpub. data). Some *M. albus* swamp eels are protogynous hermaphrodites and change naturally from female to male, supposedly in response to environmental cues (*24*).

Farmed and wild *M. albus* eels in Asian countries are reported to have a high prevalence of infection with *G. spinigerum* nematodes (*4*–*8*). This nematode is native to Asia and the most commonly reported cause of gnathostomiasis in humans in Asia (*6*,*25*). Species of *Gnathostoma* have a 3-host life cycle. Cyclopoid copepods act as first intermediate host and consume stage 2 larvae (L2) that develop into early L3 in the copepod’s hemocoel. The copepod infected with the early L3 is then consumed by second intermediate hosts such as freshwater or saltwater fish, amphibians, reptiles, or birds, in which it migrates from the stomach into other organs (most commonly the liver and striated muscle) where it develops to AL3. Felids and canids are typical definitive hosts (*7*). Humans become infected by consuming raw or undercooked meat from second intermediate hosts. Once in the human host, AL3 do not develop further, but continue to migrate through tissues, including subcutaneous spaces, visceral organs, and the central nervous system (*26*).

As many as 13 species of *Gnathostoma* are currently recognized as valid (*27*). Although it has been hypothesized that all species of *Gnathostoma* can infect humans, only 6 species have been reported to infect humans: *G. binucleatum*, *G. doloresi*, *G. hispidum*, *G. malaysiae*, *G. nipponicum,* and *G. spinigerum* (*27*). These zoonotic species use a variety of animals as definitive hosts: cats (*G. binucleatum* and *G. spinigerum*), pigs (*G. doloresi* and *G. hispidum*), rats (*G. malaysiae*), weasels (*G. nipponicum*) and dogs (*G. spinigerum*). Four species of *Gnathostoma* have been reported from wildlife in the United States. Among the 4, *G. procyonis* (raccoons) is widely distributed in the United States, whereas *G. turgidum* (opossums), *G. miyazakii* (otter), and *G. socialis* (mink) have patchy distributions (*27*).

To determine whether imported *M. cuchia* swamp eels were infected with *Gnathostoma* spp., we examined live eels obtained from various ethnic food markets in 3 major metropolitan areas in the eastern United States. We also examined individual wild *M. albus *eels, from populations introduced into open waters in Florida and New Jersey, for the presence of AL3 to determine their ability to host endemic or introduced *Gnathostoma* spp.

## Materials and Methods

### Fish Sampling and Examinations

Asian swamp eels examined for *Gnathostoma* spp. infection included 47 specimens from 5 ethnic market in 3 major metropolitan areas in the eastern United States and 67 wild-caught specimens from 4 of the 5 known introduced populations established in the continental United States (Table 1). All market specimens identified as *M.* (*Amphipnous*) *cuchia* eels purchased live from ethnic food markets during 2010–2012 included the following: 1) 10 specimens obtained from 3 markets in New York’s Chinatown in Manhattan; 2) 12 specimens from a single market in the Atlanta, Georgia, area; and 3) 25 specimens from a single market in the Orlando, Florida, area. On the basis of species identification and information in US Fish and Wildlife Service Law Enforcement Management Information System (USFWS-LEMIS) live-animal shipment records, we concluded that all or most of the market *M. cuchia* eels likely originated in Bangladesh and were shipped by air to the United States.

**Table 1:** Summary information on live Asian swamp eels from market and wild populations in the United States examined for larval stages of *Gnathostoma* spp. in 47 *Monopterus cuchia* swamp eels purchased from 5 ethnic food markets and 67 wild-caught *M. albus* (clades A, B, and C) from 4 introduced populations*

Sources and eel identifications (dates sampled)	No. samples	Total length, mm, min/max, (mean)	Body weight, g, min/max (mean)	No. eels (%) infected with *Gnathostoma *spp.	Eel specimen, parasite species, intensity, and tissue infected
Market samples: all *M. cuchia*					
New York Chinatown, 3 markets (2011 Aug 22)	10	631–850 (707)	208–693 (359)	3 (30): *G. spinigerum*	Mc 28 Gs 1K, 1M
					Mc 30 Gs 1G
					Mc 32 Gs 1M
Orlando, Florida, 1 market (2011 Jan 27, Oct 17, Oct 31; 2012 Jan 9)	25	546–781 (669)	173–565 (350)	5 (20): *G. spinigerum*	Mc 17 Gs 4L
					Mc 21 Gs 1G
					Mc 37 Gs 1L
					Mc 58 Gs 1G, 2M
					Mc 59 Gs 2G, 1M
Atlanta, Georgia, 1 market (2010 Oct 25)	12	663–825 (730)	316–796 (486)	5 (41.7): *G. spinigerum*	Mc 3 Gs 1G
					Mc 9 Gs 12L, 2M
					Mc 10 Gs 1L
					Mc 11 Gs 1L
					Mc 12 Gs 2L
All market samples (2010–2012)	47	546–850 (692)	174–796 (386)	13 (27.7): *G. spinigerum*	
Wild population samples					
Florida,Tampa area: *M. albus* clade C (2011 Nov 29–30)	14	140–912 (347)	5–693 (95)	3 (21.4): *G. turgidum; G. lamothei*	Ma 48 Gt 4M
					Ma 49 Gt 1M
					Ma 54 Gl 1K
Florida, North Miami area: *M. albus* clade C (2012 Feb 6)	11	292–710 (522)	22–343 (168)	0	
Florida, Homestead area: *M. albus* clade B (2012 Mar 10 & 12)	23	230–650 (431)	6–309 (91)	0	
New Jersey: *M. albus* clade A (2012 Apr 18)	19	190–630 (314)	4–192 (35)	0	
All wild population samples (2011–2012)	67	140–912 (395)	4–693 (89)	3 (4.5): *G. turgidum; G. lamothei*	

*Min, minimum; max, maximum; Mc, *M. cuchia*; Gs, *G. spingerum*; K, kidney; M, muscle; G, gut; L, liver; ND, not determined; Ma, *M. albus*; Gt, *G. turgidum*; Gl, *G. lamothei*.

Wild-caught swamp eel specimens collected during 2011–2012 were members of the *M. albus* species complex and included 3 geographically disjunct populations in peninsular Florida and 1 in New Jersey. Each wild population is a distinct clade (20; L.G. Nico, unpub. data). Populations and sites sampled included the following: 1) Tampa area population (clade C), 14 specimens from 2 sites in the Frog Creek drainage, Tampa Bay Basin, in Manatee County (near 27°35′18′′N, 82°30′35′W and 27°35′20′′N, 82°32′28′′W); 2) North Miami population (clade C), 11 specimens from 2 sites in the Snake Creek Canal (canal C-9) drainage, Broward and Dade counties (near 25°58′36′′N, 80°13′46′′W and 25°57′36′′N, 80°12′18′′W); 3) Florida Homestead population (clade B), 23 specimens collected from canals C-111 and L-31N, Dade County, near Everglades National Park (near 25°30′19′′N, 80°33′35′′W and 25°23′14′′N, 80°33′29′′W); and 4) New Jersey population (clade A), 19 specimens from Silver Lake in Gibbsboro, Camden County (near 39°50′21′′N, 74°57′44′′W). All sampled sites were inland, freshwater systems, and eels were collected in stream, canal, and lake habitats by using electrofishing gear.

Within 1–3 days of purchase or collection, swamp eels were transported to the US Geological Survey facility in Gainesville, Florida, where groups of <10 live swamp eels from each sampled population (i.e., market source or wild population) were held in large, clean indoor fiberglass tanks (120 cm long × 60 cm wide × 60 cm high) in 15 cm of noncirculating water from a tap source. Water in holding tanks had a pH of 7, salinity of 0.2 ppt, and temperature or 24–31°C. Large eels were separated from small eels to prevent cannibalism. Captive swamp eels held for more than several days were intermittently offered live commercially-raised earthworms (*Lumbricus terrestris*) as food, although few individuals fed on the worms. After a holding period from 1 day to several weeks, small numbers of swamp eels (1–9 individuals) were shipped live at selected intervals from November 2010 through May 2012 by overnight courier to the US Geological Survey, National Wildlife Health Center, where they were immediately euthanized with a solution of MS-222 (475 mg/L water) (tricaine methanesulfonate; Sigma, St. Louis, MO, USA) Each eel was then assigned a unique identifier code and the specimen’s total length (TL) from tip of snout to posterior end of tail was measured to the nearest mm, and weighed to the nearest gram..

Eels were decapitated, then skinned and filleted; all muscle was removed and liver, kidney, and gastrointestinal tracts were removed and separated. All organs were examined for AL3 stages of *Gnathostoma* spp. by using a dissection stereomicroscope (magnification ×4–7) then placed in a commercial grade food blender in a 5% pepsin hydrochloric acid solution and macerated. Tissue digest solution was placed in a shaking hot water bath at 37°C for overnight up to 24 hours. All digested samples were centrifuged at 3,000 *g;* solid residue was then rinsed in phosphate-buffered solution, and residue was examined for AL3 with a dissection stereomicroscope (magnification ×4–7).

### Fixation and Morphologic Identification of Nematodes

A subset of AL3 were fixed in toto in warm, 10% neutral-buffered formalin and then stored in 70% ethanol with 0.5% glycerin for morphologic identification. For all other AL3s, the posterior 2/3 of the worm was excised and fixed in cold molecular grade 100% ethanol and stored at 4°C for up to 5 months for subsequent DNA extraction and sequencing. The remaining anterior portions were fixed for whole mounts as before. Morphologic identifications were made on the basis of published keys (*7*,*28*,*29*).

**Figures 1:**
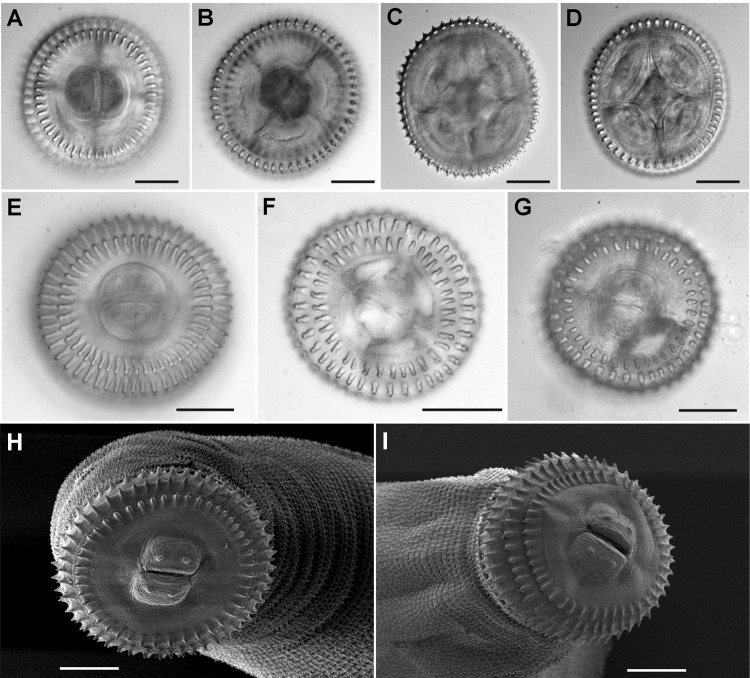
A–D) Views showing the technique used for hook counts of *Gnathostoma* spp., United States, En face (panels A, B) and posterior (panels C,D) views showing the technique used for hook counts; specimen shown here is of *Gnathostoma spinigerum* from eel 59 specimen b from gastrointestinal digestion. E–G) En face mounts of the cephalic bulbs of specimens identified as 3 different species on the basis of molecular data: panel E, specimen eel 59 G, a, *G. spinigerum*; panel F, specimen eel 48 M, c, *G. turgidum*, and panel G, specimen eel 54 K, a, *G. lamothei*. Note the difference between the hook counts in row 1 between *G. spinigerum* and the 2 other species (Table 2). H–I) Scanning electromicrograph of specimens from eel 9, identified as *G. spinigerum* on the basis of cephalic bulb hook counts. Scale bars = 50 µm.

To obtain cephalic bulb hooklet counts, we placed cephalic bulbs in a 20% ethanol, 2% glycerin solution in which bulbs were severed and oriented in an en face position in a drop of the same medium and placed under a coverslip. Hooklets on the first 2 rows (Figure 1, panels A, B) were counted by using an Olympus BX 51 microscope with brightfield and Nomarski DIC optics (Olympus Corp., Center Valley, PA, USA). The cephalic bulb was reoriented with the lips facing down for counting the 3rd and 4th rows (Figure 1, panels C, D). Images were captured digitally.

For scanning electron microscopy, the anterior portions of roundworms were post-fixed in osmium tetroxide in phosphate buffer, dehydrated through a graded ethanol series, and infiltrated with hexamethyldisilizane, following which the hexamethyldisilizane was allowed to evaporate off the specimens that were then mounted on stubs, sputter coated with gold, and scanned by using a Philips XL-20 Scanning Electron Microscope (Philips, Andover, MA, USA). Images were captured digitally.

### DNA Sequencing and Analysis

DNA was extracted following the Animal Tissue Protocol using QIAGEN’s DNeasy Blood & Tissue Kit (QIAGEN Inc. Valencia, CA, USA). Primers, NEWS2 (forward) 5′-TGTGTCGATGAAGAACGCAG-3′ and ITS2-RIXO (reverse) 5′-TTCTATGCTTAAATTCAGGGG-3′ were used to amplify a 600-bp fragment of the 5.8S rRNA gene and the intergenic transcribed spacer 2 (ITS-2) by using PCR (*30*) to corroborate morphologic identifications. Five microliters of the reaction mixture was examined by 1% agarose gel containing 0.0001% Gel Red (Phenix Research Products, Candler, NC, USA) by gel electrophoresis. Primers and nucleotides were removed from the PCR products by using ExoSAP-IT for PCR Product Clean-Up (Affymetrix, Santa Clara, CA, USA) as specified in manufacturer’s instructions. PCR products were sequenced at the University of Wisconsin – Madison Biotechnology Center’s DNA Sequencing Facility using the BigDye Terminator v3.1 (Applied Biosystems, Foster City, CA, USA) DNA sequencing system. Reaction products were analyzed by using an Applied Biosystems 3730xl automated DNA sequencing instrument. Sequences were examined with Finch TV 1.4.0 (Geospiza, Inc., Seattle, WA, USA; www.geospiza.com) and manually edited. A total of 23 individual worm sequences from 11 eels were available for analysis. These sequences were aligned along with 20 sequences of *Gnathostoma* spp. available on GenBank: *G. spinigerum*, *G. binucleatum*, *G. hispidum*, *G. nipponicum*, *G. miyazakii*, *G. lamothei*, *G. doloresi*, and *G. turgidum* from NCBI Database using ClustalW version 5.1 in MEGA (*30*) and manually trimmed to remove overhang. Sequences from this study were deposited in GenBank under accession nos. KF648531–KF648553.

Molecular analyses were conducted with MEGA version 5 (*31*). Cluster analyses were performed by using the unweighted pair group method with arithmetic mean (UPGMA) (*32*) and neighbor-joining algorithms. Statistical support for groupings was estimated by using bootstrap analysis.

## Results

All 47 market swamp eels (*M. cuchia*) examined were adult-sized (Table 1). The 67 wild-caught specimens (*M. albus*) examined included juveniles and adults. Based on results from an age growth study conducted on *M. albus* swamp eels from a subtropical lake in China (*33*), sizes of swamp eels in current study corresponded to estimated ages ranging from <1 year (eels <200 mm TL) to ≥5 years (eels >500 mm TL). 

Thirty-six AL3 of *G. spinigerum* roundworms were recovered from 13 (27.7%) *M. cuchia* swamp eels purchased from markets (5 from an Atlanta market; 5 from Orlando markets; 3 from New York markets). Five AL3 of *G. turgidum* roundworms and 1 AL3 of *G. lamothei* roundworms were collected from 2 (2.9%) and 1 (1.4%) of the *M. albus* eels obtained from wild populations in the Tampa, Florida, area. All AL3 were live and found in the digest residues of the following tissues: 21 in livers, 12 in muscles, 5 in gastrointestinal tracts and 4 in the kidneys. Only 1 eel had grossly visible white nodules on the liver, which contained AL3 of *G. spinigerum*. Among wild *M. albus* populations, gnathostomes were only collected from swamp eels from the Tampa population (Table 1).

All AL3 of *G. spinigerum* were found in imported eels from the markets, whereas *G. lamothei* and *G. turgidum* were only found in introduced wild swamp eels from open waters in the Tampa area of Florida. Matching the molecular data to the cephalic bulb hooklet counts (Table 2) corroborated that AL3 of *G. spinigerum* could be readily distinguished from *G. turgidum* and *G. lamothei* by the higher number of hooks in the first row (Figure 1, panels E–I). AL3 of *G. turgidum* and *G. lamothei* showed overlap of hook number in rows 1–3, but the 2 species could be distinguished by the fewer hooks in the 4th row of *G. lamothei*.

**Table 2:** Hooklet numbers from the 4 rows of the cephalic bulbs of AL3 *Gnathostoma* spp. and corresponding GenBank sequences collected from live Asian eels from market and wild populations in the United States*

Eel species, individual no., collection site,† tissue infected‡, AL3	Row 1	Row 2	Row 3	Row 4	Species identification and accession no.
Mc 3 G	46	46	49	51	*G. spinigerum* KF648531
Mc 9 L, a	49	50	ND	ND	*G. spinigerum* KF648532
Mc 9 L, b	48	53	55	56	*G. spinigerum* KF648533
Mc 9 L, c	42	45	43	52	*G. spinigerum* KF648534
Mc 9 L, d	43	44	47	49	*G. spinigerum*
Mc 9 L, e	43	49	50	52	*G. spinigerum*
Mc 9 L, f	45	49	50	52	*G. spinigerum*
Mc 9 L, g	43	44	49	51	*G. spinigerum*
Mc 10 L	42	47	48	54	*G. spinigerum*
Mc 11 L	40	44	50	44	*G. spinigerum*
Mc 12 L	44	41	45	49	*G. spinigerum*
Mc 17 L, a	45	43	45	50	*G. spinigerum*
Mc 17 L, b	44	48	49	50	*G. spinigerum*
Mc 17 L, c	41	44	47	49	*G. spinigerum*
Mc 17 L, d	ND	ND	ND	ND	*G. spinigerum* KF648535
Mc 17 K	43	44	44	46	*G. spinigerum*
Mc 21 G	42	45	47	52	*G. spinigerum* KF648536
Mc 26 M	33	39	40	53	G. spinigerum
Mc 28 K	43	47	50	54	*G. spinigerum* KF648552
Mc 28 M	42	43	45	46	*G. spinigerum* KF648551
Mc 30 G	40	43	42	47	*G. spinigerum* KF648550
Mc 32 M	44	45	50	–	*G. spinigerum* KF648553
Mc 37 L	42	47	50	54	*G. spinigerum* KF648549
Mc 58 M, a	ND	ND	44	52	*G. spinigerum* KF648542
Mc 58 M, b	46	49	51	52	*G. spinigerum* KF648540
Mc 58,G, c	45	47	50	52	*G. spinigerum* KF648541
Mc 59 G, a	47	48	47	52/53	*G. spinigerum* KF648538
Mc 59 G, b	48	50	52	55	*G. spinigerum* KF648539
Mc 59 M, c	42	46	47	52	*G. spinigerum* KF648537
Mean ± SD	41.8 ± 3.2	45.9 ± 3.0	47.6 ± 3.3	51 ± 2.9	*G. spinigerum* (this study)
Mean	42.9 ± 2.4	44.3 ± 2.0	44.9 ± 3.4	49.0 ± 2.9	*G. spinigerum*†
Ma 48 M, 2a	35	37	37	44	*G. turgidum* KF648547
Ma 48 M,1a	36	34	37	42	*G.turgidum* KF648548
Ma 48 M, b	32	39	40	48	*G. turgidum* KF648546
Ma 48 M, c	37	40	41	45	*G. turgidum* KF648545
Ma 49 M, a	35	37	36	42	*G. turgidum* KF648544
Mean ± SD	35 ± 1.9	37.4 ± 2.3	38.2 ± 2.2	44.2 ± 2.5	*G. turgidum* (this study)
Mean ± SD	30.8 ± 2.8	34.0 ± 2.4	36.7 ± 3.6	39.6 ± 2.7	*G. turgidum*‡
Ma 54 A,K	36	38	36	36	*G. lamothei* (this study) KF648543

*AL3, advanced larval stage 3; Mc, *Monopterus cuchia*; G, gut; L, liver; ND, not determined; K, kidney; M, muscle; Ma, *M. albus*. Letters after tissue type indicate that multiple larvae were found in 1 sample. †See (*29*). ‡See (*28*).

**Figure 2:**
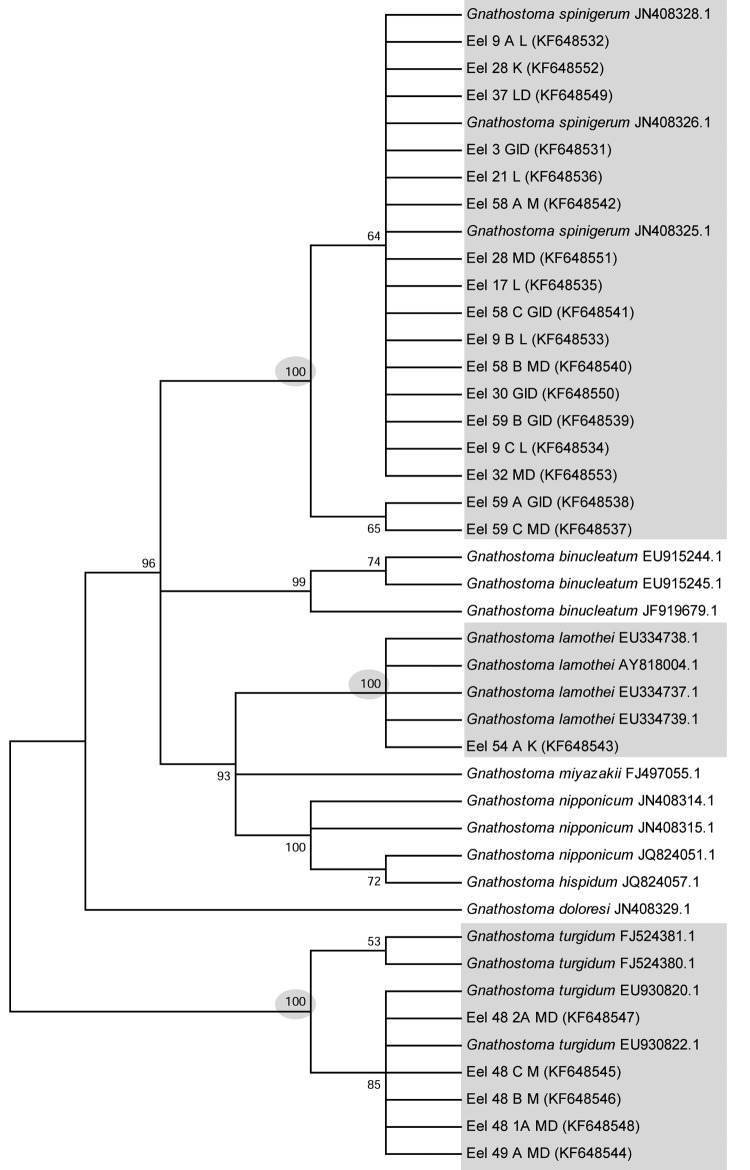
Dendogram showing the condensed bootstrap consensus tree (1,000 replicates) produced by neighbor-joining analysis for *Ganthostoma* spp. Partitions reproduced in <50% bootstrap replicates are collapsed. The percentage of replicate trees in which the associated taxa clustered together in the bootstrap test (1,000 replicates) is shown next to the branches. The sequences from the gnathostome larvae analyzed in this study fall within 3 distinct clusters (gray shading) corresponding to 3 species, with high nodal support (100%).

Assembled sequences of the amplicons varied from 511 to 659 bp. Mapping sequences to a reference sequence of *G. spinigerum*, (GenBank accession no. AB181155) comprising partial 18S, entire ITS-1, 5.8S, ITS-2, and partial 28S regions, demonstrated our sequences (see Figure 2 for accession nos.) comprised partial 5.8S, entire ITS-2 and partial 28S regions of the rRNA. After alignment and trimming, the resulting aligned database used for UPGMA and neighbor-joining analyses comprised only the ITS-2 region. Sequences from the aligned file mapped to a region between bp 1415 and 1790 on the reference sequence (AB181155), i.e., entirely within the reported ITS-2 region (Figure 2; Table 2). The UPGMA and neighbor-joining analyses resulted in the 23 isolates from this study falling into 3 distinct clusters, each representing a distinct species of *Gnathostoma*: *G. spinigerum* (17 isolates), *G. lamothei* (1 isolate), and *G. turgidum* (5 isolates) with high nodal support. Only the neighbor-joining tree is shown.

## Discussion

During 2005–2008, more than 1 billion live animals were legally imported into the United States for food and pet trade markets (*34*). Possibly beginning in the 1990s, large numbers of live Asian swamp eels were shipped from several different countries in Asia to US ethnic food markets (*9*). Through a series of Freedom of Information Act requests, we obtained USFWS-LEMIS shipment records for live animal imports for July1996–February 2010. Of the 815,000 imported live swamp eels, >95% were listed as originating from wild populations, not aquaculture sources. LEMIS provides records for wildlife shipments transported through 18 ports especially designated for such commerce. In their countries of origin (e.g., Thailand, Vietnam, China, Cambodia), swamp eels are commonly consumed as food by humans and widely available in food markets. In their native ranges, swamp eels and various other Asian freshwater fishes are infected with *Gnathostoma* spp. larvae (*8*,*35*). Increased global trade of live fish increases the risk that gnathostomes and other fish-borne parasites will be introduced into regions along with their introduced hosts.

Gnathostomiasis is a major foodborne parasitic zoonosis and a notable public health problem in areas where raw or undercooked freshwater fish are consumed by humans. Most human infections are in Southeast Asia, especially in Thailand. Infected persons can exhibit intermittent migratory subcutaneous swellings, which often recur over several years because of larval migrans. In some instances, larvae migrate into deeper tissues, causing visceral gnathostomaisis, which can be fatal if the larvae invade the central nervous system (*7*). Because human gnathostomiasis has been documented with increased frequency in countries where the parasite is not endemic, it is currently regarded as an emerging imported disease (*26*). Travel to a gnathostone-endemic area (within the past 10 years) and consuming raw or undercooked fresh water fish, frogs, poultry, or shellfish are key criteria used in diagnosing gnathostomiasis (*26*,*36*).

Our data show that live swamp eels imported to the United States from gnathostome-endemic areas could serve as a source of infection to humans in the United States. Therefore, travel outside of the United States to gnathostome-endemic areas may be limiting as a criterion in diagnosis. On rare occasions, an autochthonous infection has been reported in the United States (*37*,*38*). Several studies report a high prevalence of *G. spinigerum* in wild or farmed swamp eels in Southeast Asia (*4*–*8*).

Our recovery of live *G. spinigerum* from live swamp eels shipped from Asian sources to the United States is not surprising because the eels can survive for long periods in transport and at the market. In addition, AL3 are hardy and can remain alive for some time after the intermediate host is dead. The larvae can also survive 9–12 days at −9 to −4°C, 1 month at 4°C, and 8–9 days in 28%–35% ethanol (*7*). Considering food safety, evisceration of eels in this study rid the carcass of most of the worms (71.4%); however, 28.5% of larvae were found in the muscle, causing a risk for the consumer of raw or undercooked meat, or meat that was not frozen sufficiently before it was eaten raw. With respect to release of *G. spinigerum* roundworms into native fish and wildlife, through disposal of offal or the actual release of live swamp eels into open waters, could facilitate parasite introduction because the US environment has all the components of the parasite’s life cycle: canines and felines that serve as definitive hosts; cyclopoid copepods that serve as first intermediate hosts; fish, amphibians, and birds that serve as second intermediate hosts; and reptiles that serve as paratenic hosts (*7*).

Past studies have documented high prevalence of *G. spinigerum* larvae in live *M. albus* eels sampled from the wild, aquaculture settings, and markets in Thailand, Vietnam, and a few other Asian countries (*8*,*35*). However, *G*. *spinigerum* worms have not been reported in *M. cuchia*, a swamp eel also native to Asia but with a different natural geographic distribution than that of members of the *M. albus* species complex. *M. cuchia* eels are native to Bangladesh, the probable source of our US market specimens. In Bangladesh, although 40% of dogs surveyed were infected with *G. spinigerum*, human gnathostomiasis is reportedly uncommon (*39*,*40*).

The recovery of *G. turgidum* and *G. lamothei* from 2 and 1 *M. albus* eels, respectively, collected in open waters of Florida demonstrates that this introduced species of eel is a suitable host for North American species of *Gnathostoma*. Although these species have not been reported to be zoonotic, it has been suggested that all species of *Gnathostoma* can most likely infect humans (*7*). The previous record of *G. turgidum* infection in the United States is from the liver of a Florida black bear (*Ursus americanus floridanus*) in 1932 (*27*). Adult *G. turgidum* worms infect species of opossum (Didelphidae) and are prevalent in Mexico and South and Central America (*27*). Frogs (*Rana zweifeli*) and mud turtles (*Kinosternum integrum*) were the main second intermediate and paratenic hosts respectively of *G. turgidum* in Mexico (*28*). Adult *G. lamothei* infections in raccoons (*Procyon lotor hernandezii*) have been described in Mexico (*27*).
